# Supplementary material for: Auraptene Mitigates Parkinson’s Disease-Like Behavior by Protecting Inhibition of Mitochondrial Respiration and Scavenging Reactive Oxygen Species
Source: Int J Mol Sci. 2019 Jul 11;20(14):3409. doi: 10.3390/ijms20143409 (PMC6679046; doi:10.3390/ijms20143409)
Supplement: Supplementary file 1 [file ijms-20-03409-s001.pdf]

Supplementary Materials

Measurement of Extracellular Acidification Rate (ECAR)

SN4741 cells (2 x 10^4 cells per well) were cultured in media containing rotenone or MPP^+ 100 μM with pretreatment of AUR 2 μM or DMSO. Then oligomycin 20 μg/ml (Sigma-Aldrich, O4876), CCCP 50 μM (Sigma-Aldrich, C2759) and rotenone 20 μM (Sigma-Aldrich, R8875) was sequentially injected to each well. Basal ECAR was analyzed by XF24 analyzer (Seahorse, MA, USA).

Western blotting

Protein extract of brain tissue including SN and striatuM and SN4741 cells were prepared using RIPA buffer consisting of 50 mM Tris–HCl pH 7.5, 1% Nonidet P-40, 0.5% deoxycholate, 150 mM NaCl, and 0.1% SDS. 10% phosphatase inhibitor and protease inhibitor cocktail (Roche, Basel, Switzerland) were added to RIPA buffer. Proteins were run on SDS-PAGE gel by electrophoresis, then transferred to the polyvinylidene fluoride (PVDF) membrane by 200 mA. The membrane was blocked by 5% skim milk and incubated with a primary antibody solution containing anti-SOD2 (Abcam, Cambridge, UK) and α-Tubulin (Santa Cruz Biotechnology, CA, USA) antibody at 4 ℃ overnight. The membrane was incubated with a secondary antibody such as anti-mouse or rabbit IgG antibody conjugated to horseradish peroxidase (Pierce Biotechnology, MA, USA) and protein band was detected using ECL solution (Thermo Scientific, MA, USA)

RNA isolation and real time PCR

Total RNA of SN4741 cells treated with MPP+ and pretreatment of DMSO or AUR and was isolated using Trizol reagent (Thermo, MA, USA) and cDNA was synthesized using M-MLV reverse transcriptase (Invitrogen, CA, USA). For real time PCR analysis, cDNA was mixed with primer pairs and SYBR mix and the reaction was conducted by a Rotor Gene 6000 system (Corbett Life Science, Venlo, Netherlands). The following primers were used in this study: SOD1, 5′-GAGACCTGGGCAATGTGACT-3′ (forward) and 5′-GTTTACTGCGCAATCCCAAT-3′ (reverse); SOD2, 5′-CCGAGGAGAAGTACCACGAG-3′ (forward) and 5′-GCTTGATAGCCTCCAGCAAC-3′ (reverse); 18s rRNA, 5′-CGACCAAAGGAACCATAACT-3′ (forward) and 5′-CTGGTTGATCCTGCCAGTAG-3′ (reverse).

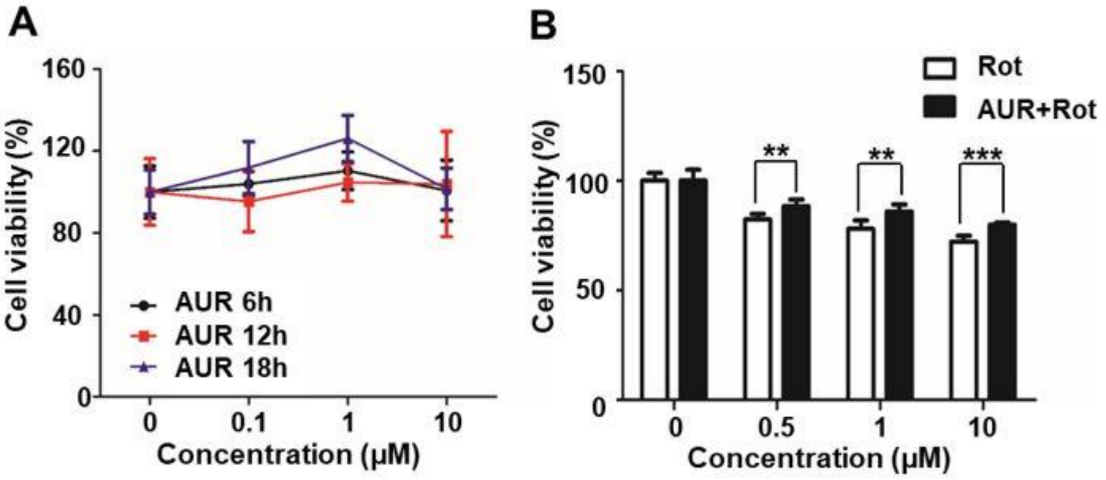

Figure S1. AUR pretreatment protects decrease of SN4741 cell viability by rotenone. (**A**) SN4741 cells were plated in 96-well-plate and incubated with or without AUR (0, 0.1, 1 and 10 μM) for increasing

time (6, 12 and 18 h). B, SN4741 cells were incubated in rotenone (0, 0.5, 1 and 10 μM) containing media for 12 hours. AUR was pretreated for 1 h. Cell viability was measured by SRB assay. Values were presented as means ± SD (**p < 0.01 vs control).

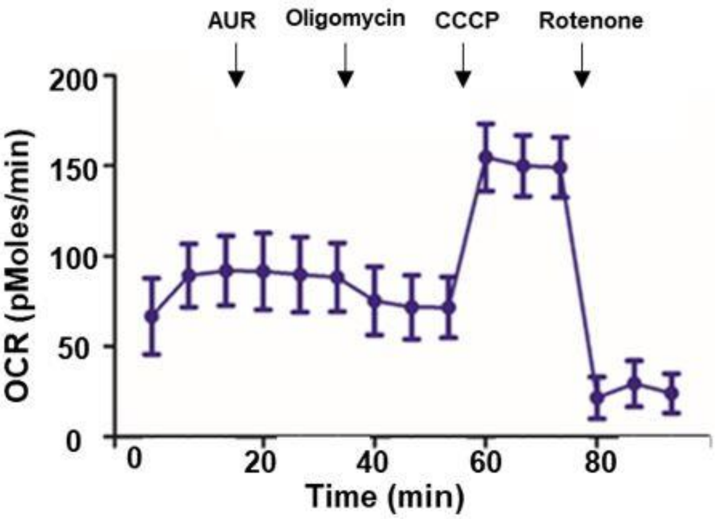

Figure S2. Short-term treatment of AUR to SN4741 had no effect on the mitochondrial respiration. OCR was measured in SN4741 cells by XF24 analyzer immediately after injection of AUR (2 μM) and it did not alter OCR.

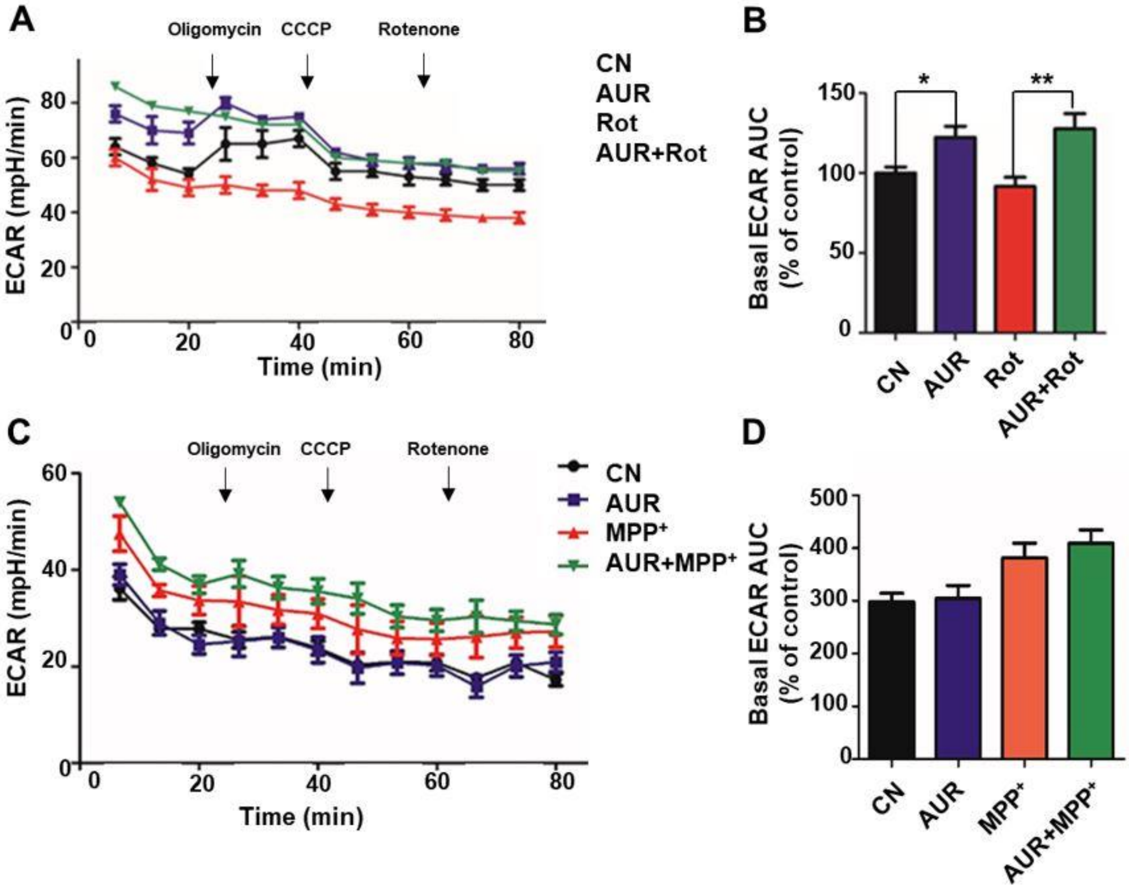

Figure S3. Pretreatment of AUR increased ECAR in the presence of rotenone and did not affect ECAR in the presence of MPP^+. (**A–D**) Basal ECAR of SN4741 cells treated with rotenone (A, B) or MPP^+ (C,D) and pretreatment of AUR or DMSO was measured by XF24 analyzer. Time point that

oligomycin, cccp, rotenone was sequentially added and is represented on the graph as an arrow. AUC of basal ECAR (B,D) was calculated by XF24 software and values were represented as means ± SD. *p < 0.05, **p < 0.01.

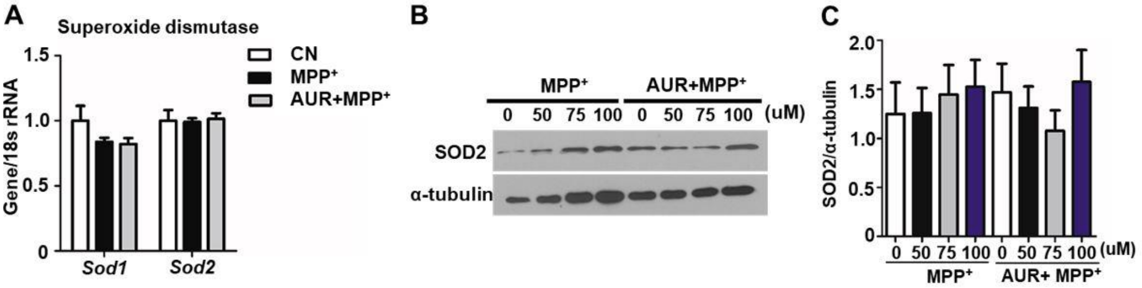

Figure S4. AUR did not alter mRNA expression of mitochondrial antioxidant enzyme. (**A**) mRNA level of Sod1 and Sod2 in SN4741 cells incubated in media containing 100 μM MPP^+ with AUR was quantified by qPCR analysis. Sod1 and Sod2 mRNA levels were normalized to 18s rRNA. (**B**) Sod2 protein level was determined by WB. SN4741 cells were treated with 100 μM MPP^+ and DMSO or AUR was pretreated for 1 h. (**C**) SOD2 protein band intensity was measured by imagej program and value is presented as mean and SD (bars).

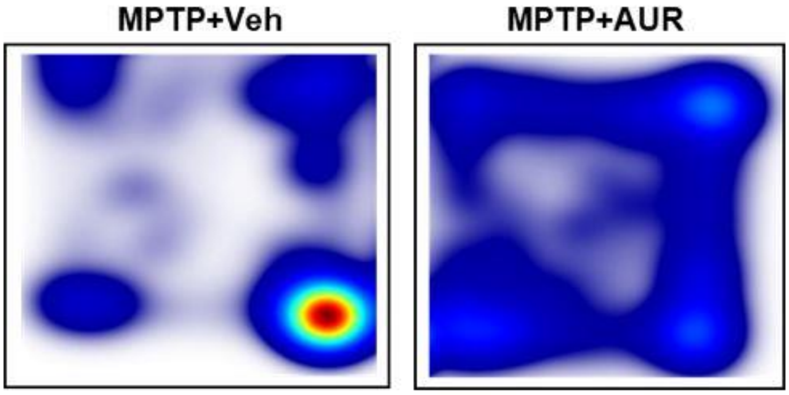

Figure S5. AUR treatment decreased time of staying in corner of MPTP-injected mice**.** MPTP (20 mg/kg) was intraperitoneally injected to male 8- week-old C57BL/6 mice. AUR (25 mg/kg) or DMSO was injected 24 h before MPTP injection and for 2 days after MPTP injection. Heat-map images were gained by EthoVision software and show decreased time in corner of MPTP-injected mice compared to AUR-cotreated mice.
